# Is routine ultrasound examination of the gallbladder justified in ICU patients?

**DOI:** 10.1186/cc9444

**Published:** 2011-03-11

**Authors:** E Evodia, I Vlachou, G Petrocheilou, A Gavala, M Pappa, L Livieratos, P Myrianthefs, L Gregorakos, G Baltopoulos

**Affiliations:** 1 Agioi Anargyroi Hospital, Athens, Greece; 2 St Paul Hospital, Athens, Greece

## Introduction

Gallbladder (GB) abnormalities are frequently seen in critically ill ICU patients. The purpose of the study was to evaluate protocolized GB US examination in medical decision-making.

## Methods

In this prospective study a twice per week GB US examination was performed in critically ill patients under mechanical ventilation (MV) for a period of 8 months independently of liver biochemistry to identify GB abnormalities. Hepatic dysfunction was defined as bilirubin >2 mg/dl and/or alkaline phosphatase >200 IU/l [1]. US findings that were evaluated included: gallbladder wall thickening, gallbladder distention, striated gallbladder wall, pericholecystic fluid and gallbladder sludge. We also recorded associated clinical and laboratory parameters: fever, WBC, MV status, liver function and administration of parenteral nutrition, analgesics, pressor agents, and predisposing factors that were associated with high incidence of acute acalculous cholecystitis (AAC).

## Results

We included 53 consecutive patients (42 males, mean age 57.6 ± 2.8 years, illness severity scores APACHE II 21.3 ± 0.9; SAPS II 53.3 ± 2.3; SOFA 10.2 ± 0.2; and mean ICU stay 35.9 ± 4.8 days) of which 25 (47.2%) had at least one US findings. Sixteen patients (30.2%) had two or more US findings. Only six patients (24%) with ultrasound findings had also concomitant hepatic dysfunction while 19 (76%) with positive ultrasound findings did not have; difference statistically significant (c^2^, *P *= 0.03). Of the remaining 19 patients, three patients had increased γ-GT only (≥150 IU/l, 415.3 ± 50.2), and two patients had increased SGPT only (≥150 IU/l, 217.5 ± 31.2). Three patients having US findings compatible with AAC underwent open cholecystectomy. Only one of them had concomitant hepatic dysfunction, as defined. Patients experiencing two or more US findings and/or liver dysfunction but not ACC were medically managed including gastric drainage, modulation of antibiotic therapy and/or interruption of nutrition until resolution of US findings or improvement in laboratory findings. In nine patients with US findings without hepatic dysfunction or increased γ-GT/SGPT, enteral or parenteral nutrition was stopped and were monitored, until improvement.

## Conclusions

Routine GB US examination was able to guide surgical therapy for AAC despite the absence of liver dysfunction. Also, it was useful to guide the medical therapy and the administration of nutrition during the ICU stay.
